# The British Orthopaedic Surgery Surveillance study: Perthes’ disease: the epidemiology and two-year outcomes from a prospective cohort in Great Britain

**DOI:** 10.1302/0301-620X.104B4.BJJ-2021-1708.R1

**Published:** 2022-04-01

**Authors:** Daniel C. Perry, Barbara Arch, Duncan Appelbe, Priya Francis, Joanna Craven, Fergal P. Monsell, Paula Williamson, Marian Knight, on behalf of the BOSS collaborators

**Affiliations:** 1 Faculty of Health and Life Sciences, University of Liverpool, Liverpool, UK; 2 Trauma and Orthopaedics Department, Alder Hey Children’s Hospital, Liverpool, UK; 3 Kadoorie Centre, Oxford Trauma and Emergency Care, NDORMS, University of Oxford, Oxford, UK; 4 North West Trauma and Orthopaedic Surgery (Cheshire and Merseyside), Health Education England, Liverpool, UK; 5 Bristol Royal Hospital for Children, Bristol, UK; 6 National Perinatal Epidemiology Unit, University of Oxford, Oxford, UK

**Keywords:** Incidence, Epidemiology, BOSS, Cohort, Perthes, Legg-Calvé-Perthes, Osteonecrosis, Avascular necrosis, Legg-calve-perthes disease, orthopaedic surgery, hips, Patient-reported outcome measures (PROMs), clinicians, randomized controlled trials, radiological outcomes, epidemiology, hip disease, cohort studies

## Abstract

**Aims:**

The aim of this study was to evaluate the epidemiology and treatment of Perthes’ disease of the hip.

**Methods:**

This was an anonymized comprehensive cohort study of Perthes’ disease, with a nested consented cohort. A total of 143 of 144 hospitals treating children’s hip disease in the UK participated over an 18-month period. Cases were cross-checked using a secondary independent reporting network of trainee surgeons to minimize those missing. Clinician-reported outcomes were collected until two years. Patient-reported outcome measures (PROMs) were collected for a subset of participants.

**Results:**

Overall, 371 children (396 hips) were newly affected by Perthes’ disease arising from 63 hospitals, with a median of two patients (interquartile range 1.0 to 5.5) per hospital. The annual incidence was 2.48 patients (95% confidence interval (CI) 2.20 to 2.76) per 100,000 zero- to 14-year-olds. Of these, 117 hips (36.4%) were treated surgically. There was considerable variation in the treatment strategy, and an optimized decision tree identified joint stiffness and age above eight years as the key determinants for containment surgery. A total of 348 hips (88.5%) had outcomes to two years, of which 227 were in the late reossification stage for which a hip shape outcome (Stulberg grade) was assigned. The independent predictors of a poorer radiological outcome were female sex (odds ratio (OR) 2.27 (95% CI 1.19 to 4.35)), age above six years (OR 2.62 (95% CI (1.30 to 5.28)), and over 50% radiological collapse at inclusion (OR 2.19 (95% CI 0.99 to 4.83)). Surgery had no effect on radiological outcomes (OR 1.03 (95% CI 0.55 to 1.96)). PROMs indicated the marked effect of the disease on the child, which persisted at two years.

**Conclusion:**

Despite the frequency of containment surgery, we found no evidence of improved outcomes. There appears to be a sufficient case volume and community equipoise among surgeons to embark on a randomized clinical trial to definitively investigate the effectiveness of containment surgery.

Cite this article: *Bone Joint J* 2022;104-B(4):510–518.

## Introduction

Perthes’ disease is an idiopathic avascular necrosis of a developing femoral head which occurs predominantly in males (at a ratio of 4:1) between four and eight years old.^1,2^ The disease usually results in long-term deformity of the hip, which is a major precipitant of premature osteoarthritis, and frequently necessitates hip arthroplasty in early adulthood.^3^ However, it is one of the most common and most poorly understood disorders encountered by paediatric orthopaedic surgeons. The incidence is believed to be approximately five per 100,000 zero- to 14-year-olds per year,^4,5^ suggesting that there are approximately 500 new cases throughout the UK annually. The origins of the disease are very closely linked to socioeconomic deprivation,^2,5-7^ though the aetiology and mechanism are so far unknown.

There is substantial variation in the treatment of Perthes’ disease, which includes observation without any intervention, admission to hospital for ‘traction’, several years of non-weightbearing in a wheelchair, months of enforced rest in abduction plaster casts, or surgery involving femoral and/or pelvic osteotomies.^8^ There are no randomized controlled trials (RCTs) to guide treatment, and only two cohort studies have sought to prospectively identify the outcomes of children with Perthes’ disease.^9,10^ There are few prognostic indicators to guide treatment; therefore, treatment is largely guided by the opinion of the treating surgeon. This has prompted the British Society of Children’s Orthopaedic Surgery and a James Lind Priority Setting Partnership to identify the treatment of Perthes’ disease as one of the top research priorities in children’s orthopaedic surgery.^11,12^

The low quality of evidence in surgery prompted the development of a framework to guide surgical research; the Idea, Development, Exploration, Assessment, Long-term study (IDEAL) framework.^13-15^ In this paper, we describe a national cohort study of children with Perthes’ disease that follows the IDEAL recommendations (IDEAL Stage 2b, ‘Exploration’). The aim was to explore the epidemiology and treatment of Perthes’ disease by investigating the disease frequency, case mix, technical intervention variables, surgeon and patient-reported outcomes, as well as safety of the patient.

## Methods

We prospectively identified a national cohort of skeletally immature individuals with a confirmed radiological diagnosis of Perthes’ disease, defined using the typical radiological features within the femoral epiphysis: flattening, sclerosis, fragmentation, collapse, and reossification. Features could be evident on either plain radiographs or MRI. Participants were eligible for inclusion at the point of diagnosis by the clinician who made the treament decisions. Exclusion criteria included prior treatment for developmental hip dysplasia (not including double nappies), prior chemotherapy, sickle cell anaemia, multiple epiphyseal dysplasia, spondyloepiphyseal dysplasia, a known coagulopathy, hypothyroidism, Gaucher disease, or a previous same-sided hip fracture.

The defined population was zero- to 14-year-olds within the geographical boundaries of Great Britain. Patients were identified between 4 April 2016 and 30 September 2017. Data were collected through a national surveillance programme, the British Orthopaedic Surgery Surveillance (BOSS) study, using a bespoke electronic platform. The full protocol detailing the mechanism of data collection has been published previously.^16^ All but one hospital in the UK that treats any hip disease in childhood agreed to participate in the study (n = 143). Patients were identified prospectively by clinical teams who, following the initial admission, completed an electronic patient record form providing the patients’ disease presentation and surgery. Patients who had been potentially missed were identified monthly by integrating reports from an independent network of trainee surgeons, and were then automatically cross-checked with known reported cases; at the same time other potential patients were identified.

Each month, an automated email was sent to nominated reporting clinicians in each hospital to ask them to verify and confirm the completeness of their monthly case uploads, including confirmation of a null report. In addition, the email highlighted potential missed patients who had been identified by the trainees. The reporting clinicians were then invited to upload the missing details or indicate a false identification. The email also identified when follow-up was due. Surgeon follow-up was at one year and two years post-diagnosis to identify outcomes, principally the radiological appearance of the hip, the need for further surgery, and the development of contralateral disease.

Patient-reported outcome measures (PROMs) were collected at a subset of hospitals recruiting patients to the study. While surgeons entered anonymous patient details to collect surgeon-reported outcomes from diagnosis, patients could be enrolled at any time during the two years of follow-up to contribute patient-reported data. The PROMs collected were the Paediatric Quality of Life Inventory (PedsQL),^17^ the youth version of the EuroQol five-dimension three-level questionnaire (EQ-5D-Y),^18^ and the Wong-Baker FACES Pain Rating Scale.^19^

The study was registered on the ISCTRN registry (ISRCTN54477575) and is reported in accordance with the IDEAL reporting guidelines.^20^ Parent representatives from the Perthes’ Association and STEPS Worldwide, the primary charitable support groups for affected children and families, coproduced the work from prefunding until publication throughout the five-year study period. The Alder Hey NIHR Young Person’s Advisory Group (YPAG), a group of children and young people involved in improving the conduct of research in children, were also involved throughout the progress of the study. The YPAG have assisted in the development of this paper and digital participant information materials, advised on conduct during interval study updates, and are engaged in the dissemination of this work to patients and the public through animations and infographics.

### Data collected

Clinicians were asked to give details regarding the following factors believed to be associated with prognosis: hip stiffness defined as ‘significantly limited abduction’ in the clinic environment; the radiological integrity of the lateral pillar;^21^ and the percentage of head involvement on the lateral radiograph.^22^ Clinicians were also asked to determine the radiological stage of disease using the modified Waldenström classification.^23^ At one- and two-year follow-up, the treatment received and outcomes were collected. The radiological outcome of ‘hip shape’ was collected if the hip was stated to be a modified Waldenström Stage 3B or beyond. The hip shape was assessed using the consensus algorithm developed by Neyt et al^24^ to describe the Stulberg classification. For hips for which follow-up radiographs were available, the roundness error on the anteroposterior (AP) radiograph was measured by an observer (DCP), who was blind to the reports from the Stulberg classification algorithm determined by participating surgeons.^25^

### Statistical analysis

The statistical analysis plan was finalized prior to data lock. Percentages were calculated, excluding missing data. Incidence rates for first presentation of Perthes’ disease (no prior diagnosis in either hip) were calculated stratified by country, region, age, and sex, using cases identified between 1 June 2016 and 31 August 2017. This interval was used allowing for a ‘run-in period’ and ‘tail-off period’ in terms of case ascertainment. Denominators were taken to be the 2016 mid-year Office for National Statistical population estimates.^26^ Poisson 95% confidence intervals (CIs) for incidence rates were calculated. The cohort’s baseline characteristics were summarized together with the initial treatment strategy. For patients with a treatment plan in place, we derived binary decision-making cut-offs most likely to influence surgical versus non-surgical strategies, using recursive partitioning. These were compared to a prior understanding of what the decision process was thought to be. We summarized the treatments received and the stage of disease of hips at one- and two-year follow-up. For hips at stages 2B or higher, we used multivariable analyses to investigate effects of baseline and treatment factors on two-year radiological outcome. For each outcome modelled, we included key known predictors specified a priori, and other baseline variables found to be associated with the outcome on univariable analysis. Roundness error was modelled using random effects linear regression, and Stulberg grade was modelled using random effects ordinal logistic regression. Risk of contralateral Perthes’ disease was estimated with the associated 95% CIs. PROMs were summarized graphically over time. Univariate analyses were used to investigate associations between baseline factors and two-year PROMs. A p-value < 0.05 was considered significant.

## Results

A total of 371 children (396 hips) had a new confirmed radiological diagnosis of Perthes’ disease identified during the 18-month recruitment period. Patients were provided from 63 NHS hospital trusts, with the median number per hospital of two patients (interquartile range (IQR) 1.0 to 5.5) over the ascertainment period. Two children (three hips) were excluded from follow-up analyses as the baseline case report forms were not completed beyond initial patient confirmation. Surgeon-reported outcomes were available in 348 of 393 hips (88.5%) at two years.

### Disease frequency

The annual incidence of Perthes’ disease was 2.48 (95% CI 2.20 to 2.76) per 100,000 zero- to 14-year-olds. There was some regional variation in incidence rates, with the highest number of patients in Northern England (Table I). A complete breakdown of the epidemiology and cohort characteristics is available in the Supplementary Material.

**Table I.** Annual incidence per 100,000 population of first presentation of Perthes’ disease.

Variable	Population, n*	First presentation	
		n	Incidence (95% CI)
All	11,311,227	304	2.48 (2.20 to 2.76)
**Region**			
**England**	9,927,566	262	2.44 (2.14 to 2.73)
London/surrounding boroughs	2,323,067	38	1.51 (1.07 to 2.07)
South	1,982,386	63	2.93 (2.25 to 3.75)
North	2,698,410	101	3.46 (2.78 to 4.13)
Central	2,923,703	60	1.89 (1.45 to 2.44)
**Wales**	523,183	16	2.82 (1.61 to 4.58)
**Scotland**	860,478	26	2.79 (1.82 to 4.09)
**Age group**			
0 to 5 yrs	4,692,365	166	3.27 (2.88 to 3.76)
6 to 10 yrs	3,850,071	117	2.81 (2.30 to 3.31)
11 to 14 yrs	2,768,791	21	0.7 (0.43 to 1.07)
**Sex**			
Male	5,793,959	231	3.68 (3.21 to 4.15)
Female	5,517,268	71	1.22 (0.96 to 1.54)

* Zero- to 14-year-olds in England, Scotland, and Wales (2016 mid-year estimate from the Office for National Statistics).^26^

CI, confidence interval.

### Cohort characteristics

The median age of the cohort was 5.4 years (IQR 4.2 to 7.4), with a strong preponderance in males (288 male (77.6%) vs 83 female (22.4%)). The ethnicity was ‘White British’ in 90.6% of patients. Among those for whom the family history was known (346 of 371 children), 20 (5.8%) had a first-degree relative affected by Perthes’ disease.

### Disease characteristics

Of the patients, 346 (93.3%) had new unilateral disease, and 25 (6.7%) had new bilateral disease. In total, 396 hips were newly diagnosed within the timeframe of the study. At inclusion, 154 hips (40.7%) were described by clinicians to have significantly limited abduction (i.e. stiffness). The radiological appearance at inclusion was Waldenström stage 2A (early fragmentation) or earlier in 301 hips (77.2%; Table II), with 262 (66.8%) having no collapse or under 50% collapse of the lateral column. The involvement of disease on the lateral radiograph was available in 249 hips (67.1%), with 154 (61.8%) reporting > 50% head involvement.

**Table II.** Stage and severity of Perthes’ disease at diagnosis.

Variable	Total
**Newly affected hips, n (%**)	
Total	396 (100)
Unilateral	346 (93.3)
Bilateral	25 (6.7)
**Documented stiffness, n (%**)	
Stiff	145 (40.7)
Minimal/no stiffness	224 (59.3)
Missing	18
**Waldenström classification, n (%)**	
0	11 (2.8)
1A	63 (16.2)
1B	134 (34.4)
2A	93 (23.8)
2B	51 (13.1)
3A	16 (4.1)
3B	15 (3.8)
4	7 (1.8)
Missing	6
**Collapse of lateral column on AP radiograph, n (%)**	
No collapse	104 (29.5)
< 50%	158 (44.8)
Exactly 50%	29 (8.2)
> 50%	62 (17.6)
Missing	4
**Head involvement on lateral radiograph, n (%)†**	
> 50% of head involvement	154 (61.8)
< 50% of head involvement	95 (38.2)
Missing	0

* Anteroposterior radiographs available for 357 hips.

† Lateral radiographs available for 249 hips.

AP, anteroposterior.

### Equipoise and decision-making

**Fig. 1:**
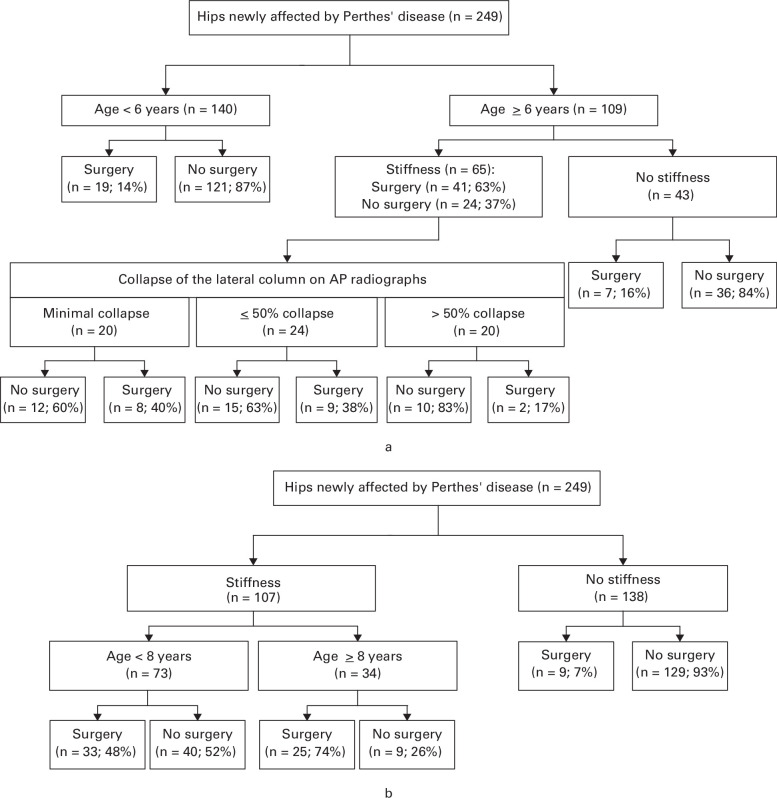
Likelihood of a surgical versus non-surgical strategy according to an a) a priori constructed decision tree and b) optimized decision tree using recursive partitioning to best fit the data, which included optimizing the cut-off for age, based on the 249 hips with a treatment strategy in place at baseline. AP, anteroposterior.

At the point of entry into the study, there was a strategy in place for treating 249 (65.0%) hips, with 67 (26.9%) planned for surgical treatment and 182 (73.1%) planned for non-surgical treatment. We hypothesized that an age cut-off of six years, hip stiffness, and the amount of femoral head involvement would be the main predictors of whether a child was planned to receive surgery (Figure 1a). However, an optimized decision tree, generated through recursive partitioning, demonstrated stiffness as the most important predictor of surgery, and an age cut-off at eight years as the next most important predictor (Figure 1b). At inclusion into the cohort, 129 (93%) hips without stiffness were planned for nonoperative treatment versus 49 (46%) of those described to have stiffness.

Over the course of follow-up, many more children underwent surgery as treatment strategies evolved. In total, 117 (33.4%) hips were treated surgically, and 233 (66.6%) treated non-surgically. The treatment details of 46 hips were unknown.

### Technical intervention variables

Of the 233 hips definitively treated nonoperatively over the two-year follow-up period, management involved observation alone in 207 hips (88.9%), with nonoperative interventions in 23 hips. Of the nonoperative interventions, 19 hips (8.3%) were advised to prevent weightbearing through the affected leg and four (1.7%) were treated in a hip spica/orthosis, with restrictions lasting for a median of 50 weeks (IQR 26 to 52).

Of the 117 hips definitively treated with surgery over the two-year follow-up period, this involved surgery to the bone in 111 (96.5%). Bone surgery most frequently concerned a ‘containment’ osteotomy: varus osteotomy (n = 63; 57.8%), shelf osteotomy (n = 35; 32.1%), Salter osteotomy (n = 5; 4.5%), and other redirectional acetabular osteotomy (n = 2; 1.8%). Other bone surgery included core decompression (n = 1; 0.9%) and hip distraction (n = 2; 1.8%), with four missing or unspecified. Soft-tissue surgery (with/without bone surgery) was undertaken in 41 of those undergoing surgery (35%), with adductor release and psoas release commonly performed.

Physiotherapy was recommended in 207 (61.2%) children. Other therapies were rare, and included botulinum toxin (n = 1), steroids (n = 4), and treatment with vitamin D (n = 5).

### Surgeon-reported outcomes

At inclusion into the cohort, 300 hips (79.1%) were described to be in early fragmentation or earlier in the disease process. At the two-year follow-up visit, 299 hips (92.0%) available for follow-up were in early reossification or later (Table III).

**Table III.** Radiological stage of hips at each follow-up timepoint.

Waldenström classification	Baseline, n	1 yr, n	2 yrs, n
0	11	7	8
1 A	63	6	3
1B	113	13	4
2A	93	30	4
2B	51	80	7
3A	14	127	72
3B	13	59	158
4	1	17	69

**Fig. 2:**
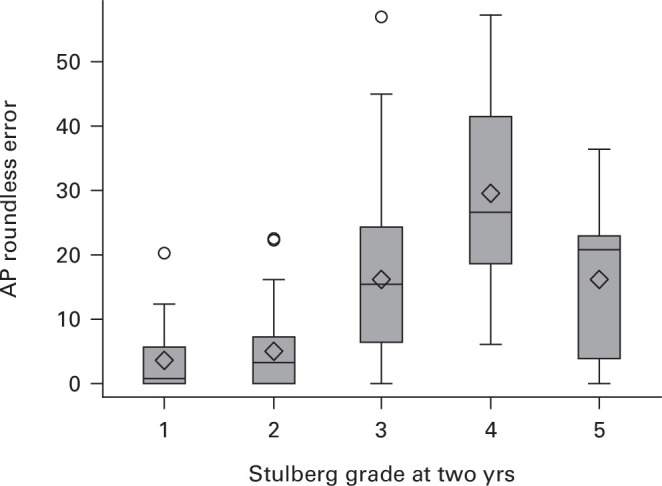
Anteroposterior (AP) roundness error stratified by two-year Stulberg grade. This was restricted to hips that reached modified Waldenström stage 3B or 4.

Overall, 227 hips (69.8%) were in late reossification or the healed stage, which was the point at which an assessment of hip shape was permitted. Of these, 39 (17.5%) were Stulberg 1 (17.5%), 59 Stulberg 2 (26.5%), 84 Stulberg 3 (37.7%), 26 Stulberg 4 (11.7%), and 15 Stulberg 5 (6.7%). A total of 141 radiographs were available to the study team. The quantifiable measure of hip shape (AP roundness error) was associated with Stulberg grades reported by reporting clinicians (Figure 2).

Ordinal regression suggests that age and sex were the only baseline factors associated with two-year Stulberg grade, with the degree of lateral pillar collapse near to statistical significance: children aged six years or more at baseline, and/or female, and/or > 50% lateral column collapse were more likely to have a worse outcome at two years. Most importantly, neither clinical stiffness nor surgical treatment on the hip appeared to have any bearing on outcome (Table IV).

**Table IV.** Multivariable ordinal regression model fitting Stulberg grade at two years with respect to baseline covariates.

Variable	Proportional adjusted OR (95% CI)	p-value
**Sex**		0.012
Male	1.00 (reference)	
Female	2.27 (1.19 to 4.34)	
**Age at entry**		0.007
< 6 yrs	1.00 (reference)	
≥ 6 yrs	2.62 (1.30 to 5.28)	
Stiffness*		0.656
None/minimal	1.00 (reference)	
Stiff hip	1.16 (0.61 to 2.20)	
**Head involvement (lateral pillar collapse**)		0.052
< 50%	1.00 (reference)	
≥ 50%	2.19 (0.99 to 4.83)	
**Surgical treatment**		0.923
No surgery	1.00 (reference)	
Surgery	1.03 (0.55 to 1.96)	

* Stiffness of hip was added to this analysis post hoc.

CI, confidence interval; OR, odds ratio.

### Patient-reported outcomes

**Fig. 3:**
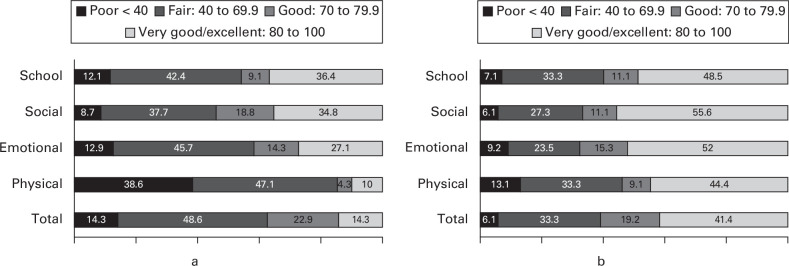
Distribution of Paediatric Quality of Life Inventory scores in Perthes’ disease patients at a) baseline (n = 70) and b) two years (n = 99), based on established categorizations.

In total, 57 of the 144 sites agreed to invite participants to complete patient-reported outcomes, and 172 participants were enrolled to provide PROMs. At baseline, 82 children were recruited with a completion rate of 85% (70/85). However, at one year and two years, completion rates were only 27% (41/150) and 58% (99/172) from 150 and 172 children, respectively. Those completing PROMs were broadly representative of the surveillance cohort in terms of age at diagnosis, sex, ethnicity, and BMI. Figure 3 illustrates the impact of the disease on the child using the PedsQL subdomains. Univariate analysis was unable to demonstrate any significant association between baseline characteristics (i.e. age, sex, BMI, stiffness, collapse, lateral head involvement, treatment, and centre-volume) and any of the recorded PROMs (i.e. EQ-5D-Y, PedsQL, or Wong Baker). See Supplementary Material for a complete breakdown of the outcome data.

### Complications

Complications of treatment, either with surgery or without surgery, were rare. There were no periprosthetic fractures associated with the surgical osteotomies. The only unplanned surgical complication was a pin exchange related to an external fixator. Within the follow-up period, 48 hips had additional surgery after their initial treatment intervention, and 26 were planned to subsequently undergo surgery. Additionally, 40 hips had undergone removal of metal hardware, with a further 14 hips scheduled to undergo removal. One patient underwent arthroplasty surgery within the follow-up period, and four were scheduled to undergo arthroplasty surgery.

Non-containment surgery completed and planned included epiphysidodesis for leg length inequality (n = 5), ablation of the greater trochanter apophysis (n = 2), realignment ostoeotomies for impingement (i.e. valgus osteotomy; n = 6), soft-tissue surgery (n = 2), femoral head decompression (n = 1), shelf osteotomy (n = 1), and osteochondroplasty for impingement (n = 1).

Seven opposite (contralateral) hips were affected by Perthes’ disease during the two-year follow-up period. The risk of contralateral disease during the two years after being diagnosed with unilateral disease was 2.1% (95% CI 0.6% to 3.6%). Given the low event rate, it was not possible to investigate the effect of baseline factors on this outcome.

## Discussion

There is widespread variation in the approaches to treatment of Perthes’ disease of the hip in children in the UK. The key uncertainty appears to be the effectiveness of ‘containment surgery’ in treating the disease. While we were able to identify the importance of known prognostic factors, we were unable to demonstrate that stiffness was an important prognostic factor, and moreover were unable to demonstrate any benefit from surgery to contain the hip.

At presentation, the strategy for the decision to offer surgery appeared to be principally based on the clinical stiffness of the hip and the age of the child. The optimized prediction algorithm demonstrated that surgeons typically used the age of eight years as a decision cut-off when planning treatment, which was different to the our hypothesized decision cut-off of six years. Treatment strategies often evolved over the course of the study, with one-quarter initially planned for surgery, yet over one-third ultimately undergoing surgery.

This is the first study to measure PROMs to demonstrate the severe impact of the disease on the child. There was a marked effect on the life of the child at diagnosis, with many aspects of health being significantly impacted even after two years. The domain of physical function was most influenced by the disease, with a marked improvement at final follow-up. However, even after two years, and despite the evident improvement in outcomes, the PedsQL scores are sufficiently impacted to be indicative of an ongoing ‘major chronic condition’, using established cut-offs of PedsQL.^27^ Notwithstanding the potential for bias among responders, there are few diseases in childhood with such a profound effect on quality of life over such a prolonged period.

The epidemiology of disease within our cohort is similar to that known, such that the disease principally affects White boys from Northern England.^4,5^ However, the incidence in our study is lower than that previously reported,^2,5,28^ and may be an underestimate of the true incidence, or may reflect a decline in frequency which has been previously observed.^5^

There are only two prior multicentre prospective studies of Perthes’ disease.^9,10^ Each concluded that surgery had a small positive influence on Perthes’ disease in specific subgroups of patients. However, each of these studies relied on post-hoc analyses, and are therefore at risk of type I error through multiple hypothesis testing and identifying subgroups through novel positive findings. We did not reproduce their findings of a positive effect of surgery, though we were able to confirm other known prognostic factors had an influence on the radiological outcome; namely age, sex, and radiological evidence of femoral head collapse.^29^

The impact of Perthes’ disease on the child has not previously been well explored. A qualitative study of children and their families explored the effects of the disease on the life of the child and wider family, identifying a range of areas of life impacted by this illness.^30^ A disease-specific core outcome set has now been formulated, which is a group of outcomes important to measure in all high-quality studies, which should usefully guide outcome choices in future research in Perthes' disease.^31^

While the BOSS study used comprehensive methods to maximize patient verification, Perthes’ disease was often managed in an outpatient setting. When investigating slipped capital femoral epiphysis, the BOSS study was able to use inpatient administrative data to identify missed cases of the disease, which was not possible in Perthes’ disease. The use of a trainee network to optimize case ascertainment, while helpful, is likely to miss patients with a childhood chronic disease, as surgical trainees are less involved in the care of these patients than other areas such as trauma. The case identification within our cohort may therefore under-represent the true incidence of Perthes’ disease in the UK.

Within the study, the radiological outcomes were determined at two years among those in the late reossification or healed stage of disease. Prior studies have demonstrated that hip shape can be reliably determined at healing, rather than waiting until skeletal maturity.^32^ However, only 227 hips (70.9%) were sufficiently mature to assign a radiological outcome at this stage. While this is unlikely to introduce bias, the missing data points do introduce uncertainties with the estimates of effect size.

PROMs at later timepoints were poorly collected within the study. Low completion rates made interpretation of the outcomes by treatment group difficult, as the study was underpowered to identify differences. We also acknowledge the potential for non-responder bias in the completion of PROMs. Nevertheless, the outcomes clearly identified the profound impact of the disease on the child. The low completion rate of PROMs probably reflected the immaturity of the national collaborative, with no previous successful prospective research in paediatric orthopaedic surgery prior to this BOSS study.

This study strengthens the argument for a definitive RCT by demonstrating sufficient numbers in the UK, revealing the far-reaching effect of the disease on the child and highlighting the uncertainties in surgical care. The definitive trial should consider incorporating several elements evident from this feasibility study. Physical function should be considered a candidate for the primary outcome, as the disease had most impact on the domain of physical function. Follow-up should be greater than two years, to enable sufficient time to ensure that the hip shape of participants can be reliably classified, and age, sex, and radiological severity should be carefully considered in the design and analysis, given the known association with outcome.

In conclusion, we have demonstrated that Perthes’ disease has a marked effect on the quality of life of children over a prolonged period. We have also shown that currently there is a widespread variation in surgical care, with significant uncertainties about the optimal treatment strategy. However, we did not find any evidence to support the view that there were improved outcomes among those undergoing surgical treatment. Therefore there is a clear need to address the question of surgery through a RCT, which now appears feasible within the context of the UK research network that has developed through the delivery of this study.

**Take home message**

- There is marked variation in the treatment of Perthes' disease, which is poorly explained, though it appears to relate to clinical uncertainties in the optimum treatment approach.

- We found no evidence to support improved outcomes among children undergoing containment surgery.
